# Distinguishing between Selective Sweeps from Standing Variation and from a *De Novo* Mutation

**DOI:** 10.1371/journal.pgen.1003011

**Published:** 2012-10-11

**Authors:** Benjamin M. Peter, Emilia Huerta-Sanchez, Rasmus Nielsen

**Affiliations:** 1 Department of Integrative Biology, University of California Berkeley, Berkeley, California, United States of America; 2 Department of Statistics, University of California Berkeley, Berkeley, California, United States of America; Aarhus University, Denmark

## Abstract

An outstanding question in human genetics has been the degree to which adaptation occurs from standing genetic variation or from *de novo* mutations. Here, we combine several common statistics used to detect selection in an Approximate Bayesian Computation (ABC) framework, with the goal of discriminating between models of selection and providing estimates of the age of selected alleles and the selection coefficients acting on them. We use simulations to assess the power and accuracy of our method and apply it to seven of the strongest sweeps currently known in humans. We identify two genes, ASPM and PSCA, that are most likely affected by selection on standing variation; and we find three genes, ADH1B, LCT, and EDAR, in which the adaptive alleles seem to have swept from a new mutation. We also confirm evidence of selection for one further gene, TRPV6. In one gene, G6PD, neither neutral models nor models of selective sweeps fit the data, presumably because this locus has been subject to balancing selection.

## Author Summary

Considerable effort has been devoted to detecting genes that are under natural selection, and hundreds of such genes have been identified in previous studies. Here, we present a method for extending these studies by inferring parameters, such as selection coefficients and the time when a selected variant arose. Of particular interest is the question whether the selective pressure was already present when the selected variant was first introduced into a population. In this case, the variant would be selected right after it originated in the population, a process we call selection from a *de novo* mutation. We contrast this with selection from standing variation, where the selected variant predates the selective pressure. We present a method to distinguish these two scenarios, test its accuracy, and apply it to seven human genes. We find three genes, ADH1B, EDAR, and LCT, that were presumably selected from a *de novo* mutation and two other genes, ASPM and PSCA, which we infer to be under selection from standing variation.

## Introduction

Most organisms harbor large amounts of, mostly neutral or nearly neutral, standing genetic variation [1]–[4]. As environments change, alleles that previously segregated neutrally, or were only weakly affected by selection, may become targets of strong selection. Examples of a change in environment that could induce such a change include invasion of a new habitat or niche through dispersal, climate changes, and introduction of novel disease agents. This type of selection, in which selection acts on already segregating alleles, is called selection from standing variation (SSV).

We contrast this model with the more commonly assumed model of selection on a *de novo* mutation (SDN). In the SDN model the selection pressure already exists when a new mutation is introduced into the population. In addition, there are several more complicated scenarios of selection. The case where an allele under selection has multiple independent origins has received particular attention [5]–[7], and is often also referred to as selection from standing variation. In this paper, we focus on the case where all copies of an allele are identical by descent, and do not consider multi-origin alleles.

Of great interest is the question of which mode of selection has been more frequent in the evolution of a species [5], [8]. In particular, if we observe a selected variant, which mode of selection is more likely to have occurred? Theoretical results by Hermisson & Pennings [5] find that SDN should be common if selection is strong and mutation rates are low, in all other cases we expect SSV to be more prevalent.

### Statistics affected by selection

Detection of selected regions has been a major goal in population genetics in recent years [9]–[13]. Rather than working with the full data, all of these studies simplified their data by using various statistics designed to detect the signal of selection (see e.g. [12], [14]). These statistics may be classified in different categories, based on the information they exploit. First, functional differences between different codon positions, and the substitution rates of synonymous and non-synonymous sites were used by [15], [16]. Another approach relies on finding related populations, where selection acts on only one of them. This leads to locus-specific high population differentiation, which may be detected by statistics such as *F*_ST_ [17] or XP-EHH [18]. A third category of statistics is based on the length of haplotypes associated with a given allele. Haplotypes associated with the selected allele will on average be younger than haplotypes carrying the derived allele, and there will therefore be fewer recombination events that break up the haplotypes. Statistics such as EHH [9] and iHS [19] were developed to detect this pattern. Finally, the site frequency spectrum (SFS) can also be used to detect departures from neutrality and hence selection. SFS based statistics usually compare various estimators of the population mutation rate *θ*. The first and perhaps most well-known statistic in this category is Tajima's D [20], but the statistic can be generalized [21], [22], and other statistics such as Fay and Wu's H [23] belong to the same family.

### Distinguishing SSV and SDN

In this study, we are interested in distinguishing the SDN and SSV models of evolution for a single putatively adaptive mutation. Barrett & Schluter [24] identify three possible ways of identifying SSV: i) the selected allele may occur in an ancestral population, ii) an allele is shown to be older than the environment it is adaptive in and iii) the signature of selection at linked loci, the selected sweep, is different between SSV and SDN. Our approach is based on differences in the genetic signature of selection, but when possible, we will compare to inferences based on i) and ii).

To understand the difference between the SSV model and the SDN model, it is important to realize that all the information regarding selection, and mode of selection, is captured by the allele frequency trajectory through time. In other words, the full allele frequency path through time would be a sufficient statistic for the selection coefficient, if it was known. As selection acts only to change the allele frequency in the selected site, and does not act directly on adjacent sites, the effects of the selection on linkage disequilibrium, haplotype patterns, allele frequencies in linked sites, etc., are only through the effects caused by the change in allele frequency of the selected allele (hitch-hiking effects). This observation is the foundation for standard population genetic theory on selective sweeps (e.g., [27]–[28]) and forms the basis for several simulation methods, in which the path of the selected mutation is first simulated and then neutral simulations are performed conditional on the allele frequency path [28]. Such simulation methods would be invalid if the allele frequency path did not contain all information regarding the selection coefficient acting on the selected mutation. Similarly, if the path of an allele is the same under the SSV and the SDN model, no additional genomic data could help us distinguish between the two models.

**Figure 1:**
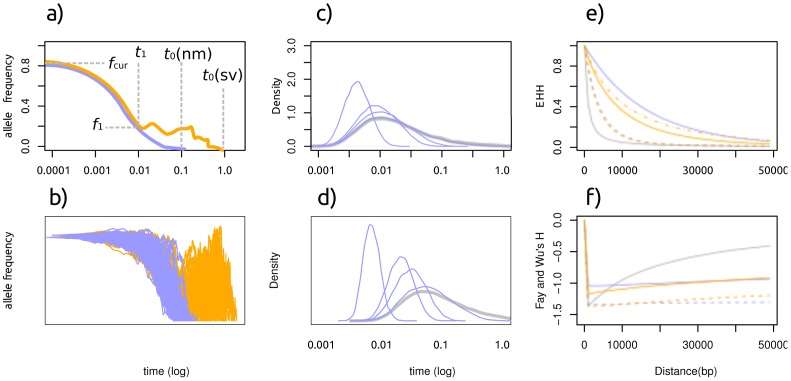
Characteristics of a selective sweep from standing variation. orange: sweep from standing variation blue: sweep from a new mutation, blue: neutral model a: A cartoon of the allele frequency trajectory with relevant parameters: *f*_1_: allele frequency at the time selection started, *f*_cur_: allele frequency at the time mutation is observed. *t*_1_: time at which selection started. *t*_0_: time when mutation arose.,. b: 100 stochastic realizations of the allele frequency trajectory. Panels c,d: Age distribution of an allele at 1% frequency and 5% frequency in a population (log scale). Blue line denotes neutrality, green lines represent selection with α = 20,100,200 and 1000 (right to left). Panels e,f: Distribution of the EHH (e) and H (f) statistic under neutrality (blue), a de novo mutation (green) and standing variation (red). Full and dashed lines represent selective pressures of α = 1,000 and 200, respectively. The dash-dot line represents α = 4000. Note that the slopes of the curves are different for the two scenarios, and the low H value around 0 under neutrality is due to the conditioning on a high frequency derived allele. Times are given in coalescent units and are plotted on a logarithmic scale.

Armed with this insight, we can further explore the differences between the two models. Figure 1a, 1b depict the trajectory, the number of copies of the selected allele through time for an SSV and SDN model. Looking backward in time, the adaptive alleles are selected at first in both models, and during this stage the two models do not differ at all. In the SSV model, however, the mutation stops being advantageous at some point in the past. Backwards from this time point, the mutation in the SSV model acts as a neutral allele, whereas the mutation in the SDN model is under selection.

As selection is the same in the phase when both alleles are selected, the difference between the models is during the phase in which selection is acting on the mutation in the SDN model but not in the SSV model. How big is this difference? It depends on two parameters: the selective advantage of the mutation under the SDN model, and the frequency of the mutation at the time when selection first start acting in the SSV model. A good measure of the difference might be the allele age distribution at this point, which is plotted in Figure 1c and 1d for a mutation at a frequency of 1% and 5%, respectively. Unfortunately, it turns out that the difference it is rather small: While the allele age of a mutation at a low frequency does depend on the selection coefficient, the difference is very small if selection is weak. Clearly, it will be much easier to distinguish between the two models if selection is strong and if the frequency of the mutation is initially high in the SSV model.

However, we cannot observe the trajectory directly, but only the diversity at linked site. It has been shown that the genetic signature of sweeps from standing variation differs in three important aspects from the signature of sweeps from new mutations [29]: at the same selection coefficient, the signal of selection from standing variation is 1) weaker and 2) affecting a narrower region. As a third difference, we expect an increased variance in both allele age and trajectory. Under the SSV model, the selected allele may be present on several haplotypes when selection starts, and these haplotypes will be affected equally strongly by selection. Thus, there will be more variation compared to SDN, and the, loss-of-diversity signal of selection will be weaker. The fact that the signal of selection affects a narrower region is due to the fact that the selected allele is older in the SSV model, and hence recombination had more time to break it up (Figure 1a, 1b). The increase in variance is evident from the large variance in the neutral phase of the allele trajectory in Figure 1b, and the wider distribution of the allele age of neutral alleles in Figure 1c and 1d. In Figure 1e and 1f we give the expected distribution of Fay and Wu's H [23] and EHH [9], two statistics used to detect selection, and where we show that the signal is indeed expected to be weaker and affecting a narrower region under the SSV model.

The objective of this paper is to develop and explore a statistical method for distinguishing between SSV and SDN models, and for providing associated estimates of relevant parameters. However, the method we develop is not intended as a new method for performing scans for selection in genome-wide data or for quantification of genome-wide levels of selection. For computational reasons, other methods might be more suitable for such genome-wide analyses. We focus on illustrating the method on a few loci previously hypothesized to be under selection in humans, but the method could as well be applied to other human loci or data from other species.

### Approximate Bayesian Computation

To exploit the characteristics of selective sweeps discussed in the previous section, we combine different statistics and calculate them for different genomic regions. Using combinations of statistics to improve inference is not a new concept, and has been applied previously (e.g. [30]). Here, we choose an Approximate Bayesian Computation (ABC) framework for combining statistics [31],[32]. ABC has the advantage that it extends naturally to allow both model choice and parameter estimates under a given model.

ABC was developed to estimate parameters of complex models in manageable computer time, and has been widely used in population genetics, most frequently to infer parameters for complex models of demographic history [32]–[37]. Several implementations of the ABC algorithm have recently been published [38]–[40], and in the past few years, various variations of the algorithm have been developed [41]–[43]. ABC is a rejection sampling algorithm used to calculate the posterior distribution of a parameter under a given model, used frequently when the likelihood cannot be calculated analytically. In ABC inference, a large number of data sets are simulated using parameters randomly drawn from a prior distribution. If a simulation does not match the observed data, it is rejected, otherwise it is retained. However, if the data is complex, the probability of a match is prohibitively low, and two important approximation steps are used: First, the data is transformed into a set of summary statistics. If these statistics are sufficient (i.e. retain all the information present in the data), this step is exact. However, in many cases, including this study, no sufficient statistics are known, and this step results in a first approximation step. In many cases, however, this transformation will still result in very low acceptance probabilities. Therefore, the condition of an exact match is relaxed. Specifically, the summary statistics based on the simulations (*S*) are compared to the summary statistics of observed data (S*). Using some distance measure *δ*, simulations are retained if |*δ* (*S*,S*)|<*ε* for an arbitrarily small distance *ε*. Frequently, some post-sampling adjustment is used in an attempt to correct for the error introduced in the second approximation step, and posterior distributions are estimated from the parameters of the retained simulations.

In this study, we propose to use ABC to distinguish between a selective sweep from a new mutation and a selective sweep from standing variation. We use simulations to determine which parts of the parameter space the method has power to make this distinction, and aim to estimate parameters under both models. We then apply our method to seven genes that were previously reported to be under selection.

## Results

### Accuracy of parameter estimates

**Figure 2:**
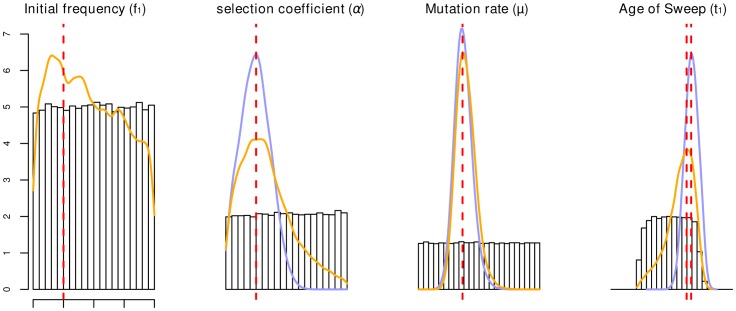
Parameter estimation accuracy under SSV and SDN model. Prior distributions are given as histograms; the orange and blue lines depict the average posterior distribution from 100 replicates of the parameters under the SSV and SDN model, respectively. The vertical dashed red line gives the parameters used for the simulation: *α* = 400, *μ* = 2.5e-8, *f*_1_ = 0.05, log(*t*_1_) = −1.51 (SSV)/−1.36(SDN). Estimates for the SSV model are less accurate for all parameters except *μ*, and 95% confidence intervals of estimates under the SSV model span the entire prior range for *f*_1_, α and *t*_1_. The age of the sweep is given in coalescence units.

We first wanted to assess how accurately we can estimate the selection coefficient and the age of the selected mutation from the SSV and SDN models. For this purpose, we performed ABC inference on simulated data sets with known parameter values. Results for a case of moderately strong selection (*α* = 400) are given in Figure 2, with *α* being the population scaled selection coefficient *α = *4*Ns*. As can be seen from the figure, the mode is an accurate estimator of the true value for both models. However, in the SSV case the posterior distribution is much broader than under the SDN model, and the 95% confidence interval extends to the edges of the prior, indicating low accuracy in the estimate. For the initial frequency parameter, *f*_1_, the posterior differs only marginally from the prior, and therefore this parameter cannot be reliably estimated.

### Accuracy of model choice

**Figure 3:**
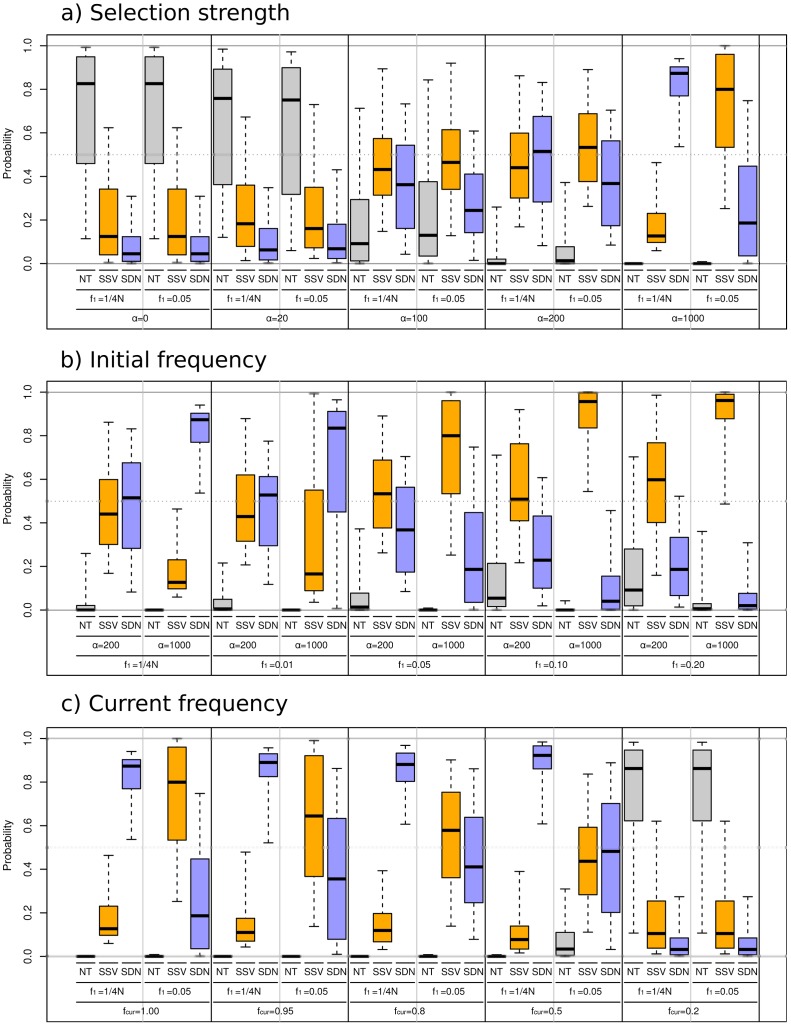
Simulation results for ABC model choice procedure. We simulated data using the fixed parameter values given in the lower part of the figure. The boxplots show the lower and upper quartiles, the median and the limits of a 95% interval of the posterior probability for the NT (blue), SSV (red) andSDN(green) models, respectively. Panel a: We compare the effect of the increasing selection coefficient *α*. Panel b: The effect of increasing initial frequency *f*_1_. Panel c: The effect of the current frequency *f*_cur_, In panels a,b *f*_cur_ was set to 0.95, and in panel c, *α = *1,000.

We aim to identify parameter regions where we can distinguish between the SSV and the SDN model. As a control, we also consider a model of neutral evolution (NT), where an allele increases to high frequency solely due to genetic drift. In particular, we are interested in three parameters that are expected to have a strong influence on model choice accuracy: the selection parameter *α*, the frequency of the mutation when it became selective advantageous, *f*_1_, and the current frequency of the selected allele *f*_cur_. In Figure 3 and Figure S1, we explore the accuracy of our model choice procedure in three series as a function of *α*, *f*_1_, and *f*_cur._.

We find (Figure 3a) that in cases where *α*<100; the method cannot reliably distinguish between selection and a neutral model. This is not surprising, as for such values of *α*, standard neutrality tests have little or no power to detect selection [44], [45]. For selection coefficients of *α* = 100 and *α* = 200 the neutral model has a very low posterior probability and would be rejected, but we still do not have sufficient power to distinguish the signals from SSV from SDN. Only under strong selection (*α* = 1,000) do we have reasonable power to distinguish between SSV and SDN. Thus, we find that there is a parameter range of α between 100 and 500, in which selection can be reliably detected, but the two models of selection are statistically indistinguishable.

In the second series (Figure 3b), we vary the initial allele frequency (*f*_1_). We find that simulations under the SSV model, with *f*_1_ = 1%, are identified as SDN models, but that the accuracy in model choice increases with *f*_1_. For larger values of *f*_1_, we can detect selection when selection is strong (α = 1,000). For high initial allele frequency (*f*_1_ = 20%) we correctly infer the true mode of selection even when α is 200. This suggests that the ability to distinguish the two models increases with *f*_1_. Furthermore, we also find a negative relationship between the estimated value of *f*_1_ for a data set and the posterior probability of the SDN model (Figures S2, S3 and S4): As we would expect, the larger the estimate of *f*_1_, the lower is the posterior probability of the SDN model, and we find a strong negative correlation (R^2^ = 0.51) between these two quantities based on 1,000 simulations.

In the third series (Figure 3c), we investigate the effect of the current allele frequency *f*_cur_ on the model comparison. For simulations under the SSV model, we find that the accuracy strongly decreases with *f*_cur_. For *f*_cur_ = 0.2, we classify slightly less than half of the data sets correctly. This is in contrast to simulations under the SDN model, where the power to correctly classify simulated data sets gradually increases with *f*_cur_. Thus, in studies aimed at detecting selection on standing variation, the false positive rate should depend only slightly on *f_cur_*, but the false negative rate is expected to increases drastically when *f*_cur_ is low.

**Figure 4:**
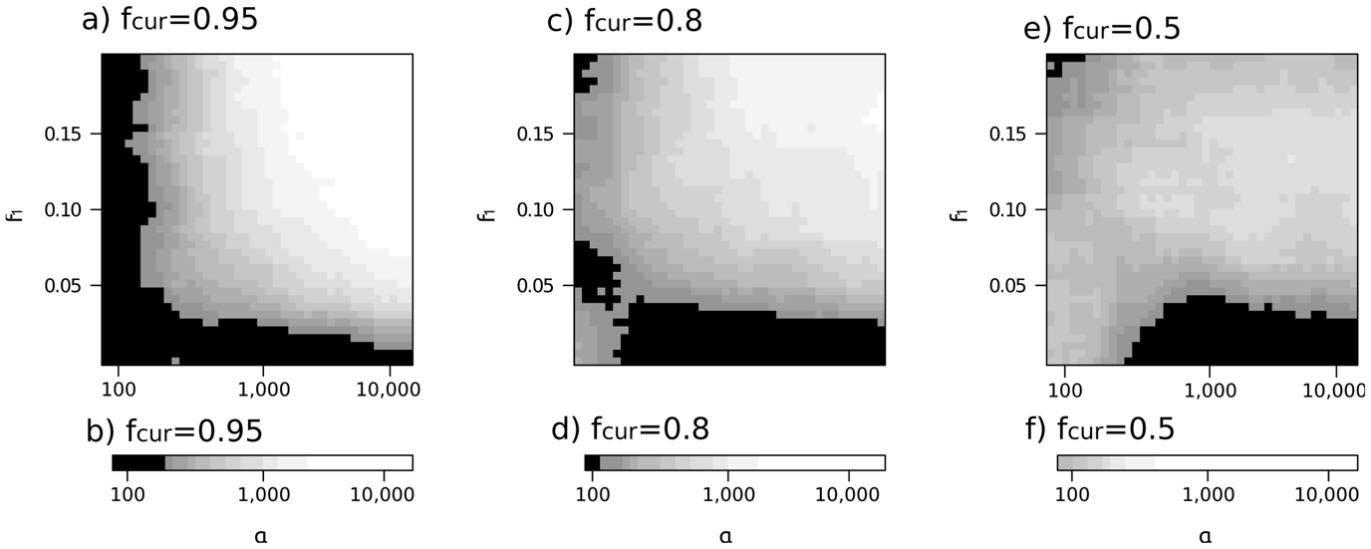
Parameter regions where distinction between models is possible. On x and y axes are the prior ranges for selection coefficient and initial frequency of a selective sweep, respectively. Panels a, c and e give simulations under the SSV model, panels b, d and f for the SDN model. The different panels represent different current frequencies: In Panels a, b *f*_cur_ is 0.95, in c, d *f*_cur_ = 0.8 and in panels e and f *f*_cur_ = 0.5. Color gives the proportion of simulated data sets that were assigned to the correct model, when compared to the two alternative models. Black areas correspond to regions where this proportion is less than 50%, white areas to parameter regions where 95% or more of the data sets are correctly assigned. Each shade of grey corresponds to a 5% increase in the number of correctly assigned data sets.

Figure 4 illustrates how the selection parameter (α) and the initial allele frequency (*f*_1_) affect the accuracy of model choice between the SSV, SDN and NT models for three values of *f_cur_* (*f*_cur_ = 0.95, *f*_cur_ = 0.8 and *f*_curr_ = 0.5). As in Figure 3, the number of correctly assigned data sets increases with *α*, *f_1_* and *f*_cur_. Under the SSV model, the gradient with which the power declines is strongest when *f*_cur_ is large (95%, Figure 4a), and becomes less pronounced for smaller *f*_cur_ (see Figure 4c and 4e). For *f*_cur_ = 95% (Figure 4a), there is a region with *f*_1_>0.05 and α>1,000 where there clearly is very high power to infer the correct model. On the other hand, for α<200 or *f*_1_<0.03, we make incorrect inferences more than half of the time, indicating that in these regions of the parameter space, the signal of the sweep is too weak to discriminate between the SSV and SDN models. While that global pattern is the same for *f*_cur_ = 0.8 and *f*_cur_ = 0.5 (Figure 4c, 4e), the distinction between regions where we can and cannot assign simulated data sets correctly is less pronounced.

Quite surprisingly, however, we find that for *f*_cur_ = 0.8, the number of correctly assigned data sets increases when selection is low. The same trend holds for *f*_cur_ = 0.5 (Figure 4e), however here the influence of selection is even weaker, and inference becomes quite ambiguous, with posterior probabilities ranging from 60% to 80% in the entire parameter space.

In contrast, the pattern is much simpler for simulations under the SDN model (Figure 4b, 4d and 4f), where the probability to correctly identify the model increases with decreasing *f*_cur_. When *f*_cur_ is set to 0.95, we need a selection coefficient of *α* = 1,500 to make confident inferences. For *f*_cur_ of 0.8 and 0.5, this value decreases to 900 and 300, respectively.

In summary, a high current allele frequency increases the power to distinguish between SSV from SDN (Figure 3c, Figure 4). The frequency with which the SDN model is correctly inferred increases slightly with decreasing *f*_cur_, presumably because the selected phase makes up a larger proportion of the trajectory.

### Applications

We illustrate our model choice procedure by analyzing seven genes that have previously been identified as candidates for being under selection. These genes are ADH1B, ASPM, EDAR, G6PD, LCT, PSCA and TRPV6. The genes were selected using the following set of criteria: i) there is evidence for selection from a previous study, ii) a putative causal mutation has been identified and iii) the putative causal site has reached a high frequency in at least one population, but has not yet reached fixation. In addition, we also analyzed four regions that were noncoding and presumably neutral. We retrieved polymorphism data from the 1000 Genomes Project low coverage data [46] using *tabix* [47]. Ancestral genotypes were inferred by comparison to the homologous chimpanzee allele. If a signal of selection was present in more than one population, we used data for the population where the selected site was most frequent, to facilitate inference. Model choice and parameter estimation were performed using the procedures described in the methods section. In contrast to the inference on simulated data sets, here we explicitly model varying recombination rates and the complex demographic history of the human population.

**Figure 5:**
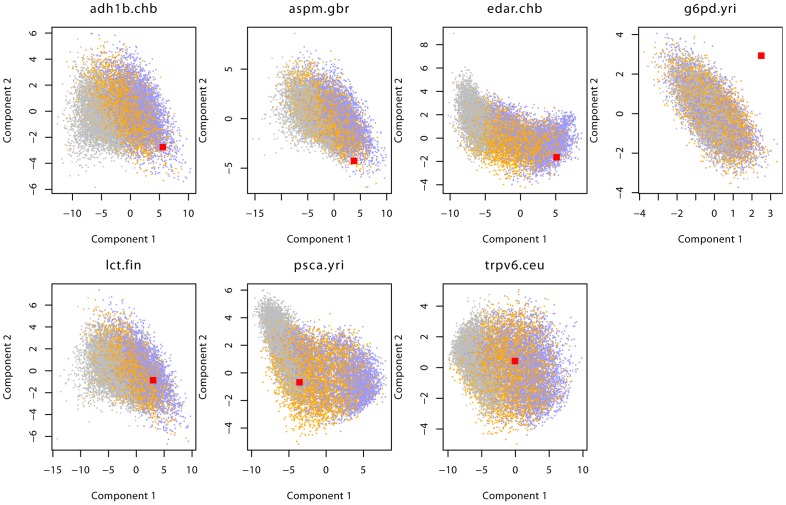
Distribution of summary statistics of 7 genes. This figure shows the observed (red) and prior predictive distribution of the first two PLS-DA components. Neutral simulations are shown in grey, SSV in orange and SDN in blue. For G6PD we show components 2 and 3 to highlight the finding that none of the three models analyzed is able to model the data for this gene.

Results for the sample genes are given in Figure 5 and Figure S4, as well as Table 1. For six of the seven genes analyzed, the neutral scenario was strongly rejected with a posterior probability of less than 1%, and we can confirm the prior evidence that these genes are under selection. Three of those genes, ADH1B, EDAR and LCT, were found to be under selection from a new mutation and one gene, TRPV6 could not be assigned with any significant probability to either model. Two genes, ASPM and PSCA, were found to be under selection from standing variation. Finally, none of the three models provided a good fit to observed data in the G6PD gene, suggesting that neither of the models is appropriate for this gene. In the following paragraphs, we will discuss each gene in some detail, and give estimates for selection coefficient and time when appropriate. All estimates are given with a point estimate for the mode, and the lower and upper bound of a 95% Highest Posterior Density interval in brackets. Estimates in years were made assuming a generation time of 25 years.

**Table 1:** Genes analyzed in this study.

					Estimates			
Gene	chr	function	pop	Model	*S*	*t*_1_ (years)	*t*_0_ (years)	References
**ADH1B**	4	Alcohol metabolism	CHB	SDN(0.78)	0.036 (0.009–0.192)	-	11,100 (1,900–42,900)	[50]
**ASPM**	1	microcephalism	GBR	SSV(0.87)	0.029(0.003–0.17)	17,400 (800–56,400)	79 (17–288) ky	[56]
**EDAR**	2	NF-κB Activation	CHB	SDN(0.88)	0.14 (0.07–0.31)	-	11,400 (4,300–43,700)	[61]
**G6PD**	X	malaria resistance	YRI	-	-	-	-	[66]
**LCT**	2	lactase persistence	FIN	SDN(0.99)	0.025 (0.004–0.20)	-	11,200 (1500–64,900)	[73], [74]
**PSCA**	8	Involved in bladder & pancreas cancer	YRI	SSV(0.86)	0.035 (0.004–0.015)	8,000 (1,000–54,900)	191 (50–698) ky	[86]
**TRPV6**	7	Calcium absorption	CEU	SSV (0.55)	0.032 (0.005–0.25)	7.600 (900–43,300)	211 (29–697)ky	[90]
				SDN (0.45)	0.023 (0.007–0.08)	-	23,400 (6,400–70,400)	

Chr: chromosome, pop: population we analyzed using the population code from the 1000 genomes project; For each gene, we give the favored model(s) and in brackets the posterior probability for that model.

## Discussion

### Applications

#### ADH1B

The ADH1B gene encodes one of three subunits of the Alcohol dehydrogenase (ADH1) protein, a major enzyme in the alcohol degradation pathway that catalyzes the oxidization of alcohols into aldehydes. ADH1B is part of a 60 kb gene cluster on chromosome 4, encoding for all three ADH1 subunits. Selection on the major ADH gene complex has received major attention as it is suggested to be one of the major genetic causes of alcoholism risk [48], and a possible cause of the “alcohol flush” phenotype prevalent in many Asian populations, where individuals turn red due to increased acetaldehyde levels in the blood after alcohol consumption [49]. As a result, the genes are well studied and several non-synonymous polymorphisms are known to have various effects on enzyme activity [50], [51]. One particular allele, Arg47His, has been proposed to be under selection based on several lines of evidence: First, the derived Histidine allele results in an increased enzymatic activity. Second, age estimates of the derived allele based on its frequency correlate with the onset of rice domestication [48], [49] and the availability of fermented beverages [52].

In our analysis, we analyzed the CHB population where the allele is found at a frequency of 0.71 in the 1000 genomes data. For this data set, we could clearly reject the neutral model, with a posterior probability of 1e-8. The SDN and SSV models have posterior probabilities of 78.3% and 21.7%, respectively, indicating slightly stronger evidence in favor of the SDN model. Under this model, we estimate a selection coefficient of *s* = 0.036 (0.009–0.19), and an age of the mutation of 11,100 (1,900–42,900) years It is remarkable that this age corresponds very well with the arrival of rice agriculture and the availability of fermented beverages in China around 10,000 year ago [49]. Our finding of evidence for a *de novo* sweep is conflicting with the fact that the derived 47His allele also occurs at a high frequency in Western Asian populations, but only at low frequencies in Central Asian and Indian populations [48], a pattern of genetic variation that has previously been suggested to be a result of selection on standing variation [48].

#### ASPM

The ASPM (abnormal spindle-like microcephaly associated) gene has been identified as a major determinant of brain size [53]. Much attention has been focused on the difference between humans and chimpanzee in that gene, and several studies [54], [55] have quantified these differences and found an unusual high amount of fixed substitutions between these two species, indicating positive selection on the branch between humans and chimps. In addition, recent ongoing selection was proposed based on the finding that a single haplotype was unusually frequent in several populations [56]. However, the interpretation of their results stirred considerable debate [57]–[60], with researchers pointing out that the haplotype distribution found by [56] is not that unusual [60] and that neutral demographic scenarios are able to produce haplotype distributions similar to the one observed in ASPM [58].

We used the non-synonymous SNP A44871G (rs41310927) for our study, which was identified in [56] as a putative causal variant in our analysis. We found evidence for selection on standing variation (Pr(SSV) = 0.87), with little support for the neutral and SDN model with posterior probabilities of 0.13 and 2e-7, respectively.

We estimate a rather weak selection coefficient of 0.029 (0.003–0.170), and estimate that selection started to act 17,412 (771–56,443) years ago, and an age of the mutation of 97 (17–289) ky. This is considerably older than the estimate of 5,800 years for the most recent common ancestor of the selected allele by Mekel-Bobrov *et al.* [56], a difference that might be due to the fact that we assume a different demographic history.

#### EDAR

The EDAR gene region has been suggested to be under selection in East Asians based on multiple genome scans [10], [11], [19] and has been studied in more detail by Bryk *et al*. [61]. EDAR encodes a cell-surface receptor that activates a transcription factor [61], [62], and, among other phenotypes, has been associated with the development of distinct hair and teeth morphologies [62], [63]. A non-synonymous SNP (rs3827760, V370A) has been associated with these phenotypes, and has been confirmed in an in vitro study to enhance the activity of the EDAR gene [61]. The rs3827760 SNP lies in a DEATH-domain that is highly conserved within mammals [18], and is found at a very high frequency in East Asian and American individuals, but is absent from all European and African populations [61].

In the 1000 genomes data, EDAR shows the strongest signal of selection for EHH, Tajima's D and Fay & Wu's H among all genes we analyzed. This is reflected in our model choice analysis, where we find a 88.5% probability that the V370A polymorphism originated from a new mutation. The probability for the SSV model was 13.3%, and the neutral model did not receive any measurable support. We estimated a very high selection coefficient of *s* = 0.15 (0.04, 0.31), and an origin of the mutant allele 3,000 (1,400, 6,900) years ago. This estimate is most likely too young, as the allele is also present in Native American population and so is strongly expected to have been present before the colonization of America. A possible explanation for this is that selection does not act codominantly on EDAR. Comparing our codominant model with a model where the dominance parameter *h* was allowed to vary between 0 and 1 resulted in a strong favor for the more complex model (Bayes Factor = 36). Under this model we estimate a selection coefficient of s = 0.14 (0.07–0.31), but a much older age of the allele of 11,400 (4,300–43,700) years. This is at the lower end of estimates for the time of colonization of the Americas [33], [64], indicating that the derived allele might have moved into the American populations at a low frequency. This hypothesis is consistent with the very high divergence of the EDAR region between the Mexican and Chinese populations, where we find an *F*_ST_ of 0.36 (excluding the conserved DEATH-domain), which is much higher than the genome-average *F*_ST_ of 0.069 between these two populations [65]. This may indicate that the 370A allele has risen in frequency largely independently between these two populations.

This is in contrast to the analysis of Bryk *et al.* [61], who estimated that the derived 370A allele has been fixed 10,740 years ago. However, both in the 1,000 genomes data and the data of [61], the site is still segregating within the CHB population. While we cannot exclude the scenario of fixation and recent reintroduction of the ancestral allele, the high divergence between Native Americans and East Asians seems to favor a more recent sweep.

#### G6PD

The G6PD gene is located on the X chromosome, and is one of the best studied cases of selection in humans [9], [66]–[68]. The G6PD gene encodes the Glucose-6-phosphate dehydrogenase protein, the first enzyme in the pentose phosphate pathway. The G6PD gene has long been associated with reduced-efficiency erythrocytes [69], [70], and several hundred variants causing various levels of reduction in catalytic activity have been discovered [71], leading to a significantly reduced fitness in affected individuals. As a benefit, however, G6PD deficiency provides resistance to malaria [72] and therefore even strongly deleterious alleles rise to considerable frequencies in populations where malaria infections are epidemic. Due to these antagonistic selective pressures, G6PD in populations affected by malaria is one of the best examples of balancing selection described in the human genome.

We use the A/A- polymorphism (rs1050828), identified by [66] as the putative site under selection. When applying our method, however, none of the models provided a good fit to the data, indicating that the models we used are too simplistic for the complicated history of G6PD (see Figure 5). The combination of summary statistics with a low EHH, very low IHS and high, non-significant values for Tajima's D and Fay and Wu's H cannot be captured by either of our models. This is not surprising given that the selection on the G6PD locus cannot be described as a selective sweep, but is the effect of balancing selection. It is encouraging the method in this case indirectly, through a poor model fit, helps determine that the simple selective sweep models considered here are not appropriate for this locus.

#### LCT

In most mammals, the ability to digest lactose, a common disaccharide in milk, decreases when they stop being milk-fed. In contrast, in many humans the main enzyme used to digest lactose into monosaccharides, continues to be expressed even in adults, a phenotype known as lactase-persistence [73]–[76]. Several presumably independent alleles have been identified that confer the same phenotype [76] in different populations. The first and possibly best-characterized allele is the C/T-13910 polymorphism (rs4988235) that is particularly prevalent in Northern European populations and has been shown in Finnish populations to be 100% associated with the lactase phenotype [77]. Further evidence that the T-13910 allele is causal for the persistence phenotype is given by *in vitro* analyses [78], [79] that found increased enhancer activity.

We analyzed the FIN population from the June 2011 data release of the 1,000 Genomes Project, using the C/T-13910 polymorphism as the selected site. We found a 98.7% posterior probability for the SDN model and only a 1.2% posterior probability for the SSV model, indicating that this particular LCT allele most likely was under selection shortly after it arose. We estimated a rather low selection coefficient of 0.025 (0.003–0.19), and an origin of the mutation 11,200 (1,500–64,900) years ago. Our estimate is much older than the estimates by Bersaglieri *et al.* [75], who estimated a selection coefficient between 0.09 and 0.19, and an age of the mutation of 1,625–3,188 years using a deterministic approximation based on the observed frequency of the allele. The fact that they used a deterministic approximation may explain the fact that we have wider confidence intervals. Our estimate is more consistent with the estimate of Tishkoff *et al*., [76] who used the width of the sweep region to date the selected allele to an age of 7,998 years and obtained an estimated selection coefficient of 0.069. Our estimates are also in good concordance with the estimate of Itan *et al.* [80]. In their study, they modeled the spread of lactase persistence through Europe using a spatially explicit ABC model, which takes advantage of the arrival of dairy farming in various locations. They estimated a selection coefficient of 0.095 in dairy farmers and a slightly older age for the selected allele (7,441 years). While all studies suggest a more recent origin of the selected allele, we note that the confidence intervals on both the selection coefficient and age of the sweep overlap between all four studies.

A complimentary approach to dating the age of an allele, and estimating selection coefficients from modern DNA data, is the usage of ancient DNA [81]–[83]. Indeed, the derived allele of the LCT the C/T-13910 polymorphism as was found in a single copy in a 5,000 year old sample from Sweden [82], and at a higher frequency of 27% in the Basque country in a sample of approximately the same age [83]. In contrast, the derived allele was absent from an Eastern European sample roughly 7,000 years old [81]. These findings are in good agreement with our estimates and other estimates on genetic data. Based on this ancient DNA evidence, it has been speculated that the LCT allele may have swept from standing variation [83], mainly due to the fact that the derived allele is found at a rather high frequency only two millennia after the introduction of agriculture in that population. However, if the allele was mostly neutral before the arrival we would expect it to be rather old, and in particular we might also expect to see the derived T allele in African populations, which is not the case. Calculating the expected age of an allele at a frequency of 27% [84], [85] results in expected ages of 480 ky and 6,500 (2,500–36,000) years for neutrality and selection, respectively, using our estimated selection coefficient and an effective population size of 10,000. While these estimates based solely on allele frequencies should be interpreted with great caution, they nevertheless show that our estimate of a *de novo* selected mutation is consistent both with the observed allele frequencies around 5,000 years ago and an assumed origin of dairy farming 11,000–12,000 years ago.

#### PSCA

The prostate stem cell antigen gene (PSCA) on chromosome 8 has been proposed to be under selection by [86] based on an analysis of population differentiation in a global array of human populations. A non-synonymous SNP in PSCA (rs2294008) is known to be involved in various forms of cancer [87], [88], and we therefore used it as the causal site in our analysis. Interestingly, the derived allele is present in all human populations although the frequency varies considerably between different populations [86]. The highest derived allele frequencies of more than 75% are reported in West African and East Asian populations, whereas some sub-Saharan African and most Native American populations have allele frequencies below 20%. This worldwide distribution of the allele was interpreted as evidence of selection from standing variation [86].

Our analysis confirmed this hypothesis based on analyses of data only from the Yoruban population, with the SSV model receiving a posterior probability of 86.0%, compared to a posterior probability of 23.9% for the SDN model, and 1.2% for a neutral model Under the SSV model, we estimate a selection coefficient of 0.035 (0.004–0.15), with selection having started 8,000 (1,000–54,900) years ago, and the allele being 191 (50–698) thousand years old. The fact that the mutation is distributed globally supports our inference of a sweep based on standing variation.

#### TRPV6

TRPV6 is in the heart of a 115 kb region on chromosome 7 that has been reported to be under selection [10], [89] and has been closely investigated by Akey *et al.* [90]. TRPV6 codes for a protein subunit that encodes cation pores, particularly for calcium ions [90], [91]. TRPV6 was found to be in a region of accelerated evolution on the human lineage, as indicated by an elevated ratio of non-synonymous to synonymous fixed differences [90]. In particular, three non-synonymous mutations segregating in humans were found, with a striking diversity pattern; the derived allele was at an intermediate frequency in all African population, but at frequencies of 90% and more in the rest of the world. In addition, both Tajima's D and Fay and Wu's H statistics were significantly negative for non-Africans and European-Americans. For this reason, we restricted our analysis to the CEU population, and used the first of the non-synonymous SNP (rs4987682) as the focal site for our analysis, as it was the only one that was in the N-terminal region of the TRPV6 protein, the suggested target of selection [90].

While the neutral model could be rejected with a posterior probability close to zero (8e-7), the separation of SSV and SDN model remained inconclusive, with posterior probabilities of 0.55 and 0.45 respectively. The estimate of the selection coefficient was very similar for both models *s*_SSV_ = 0.032 (0.005–0.25), *s*_SDN_ = 0.023 (0.007–0.08), but the confidence interval is much smaller under the SDN model, as expected. Furthermore, the estimated age of the allele differed between models: Under the SSV model, the mutation is inferred to be 211 (29–697) ky old, but became selected only 7,600 (900–43,300) years ago. Under the SDN model, the mutation arose and became selected 23,400 (6,400–70,400) years ago. These findings are in good concordance with the patterns of diversity found previously [10], [90], and in particular the evidence that the signature of selection is shared between all non-African populations and thus selection started likely less than 100,000 years ago. Also, the estimate under the SSV model that selection started less than 10,000 years ago is concordant with the role of TRPV6 in absorbing calcium [90].

#### Neutral regions

In addition to these genes, we also analyzed four putatively neutral regions that were 5 Mb away from our candidate genes. This distance should be big enough that the neutral region are not impacted by the selective sweep, but are likely influenced by the same mutational processes as the selected regions. For all these regions, the neutral model had the highest posterior probability, with posterior probabilities of 0.758, 0.932, 0.994 and 0.999 for the four regions. This indicates we are indeed able to discern selected from neutral regions.

### Conclusions on data applications

The distribution of summary statistics in Figure S5 illustrates the impact of choice of summary statistic for model inference [92]. Very high values of EHH are clearly indicative of the SDN model, at both a 10 kb and 20 kb distance. Both the SSV and SDN models are associated with low IHS values, whereas the neutral regions have IHS values closer to zero. Tajima's D and Fay and Wu's H are both very informative for model comparison, with SDN genes having very low D values, SSV genes having D values close to zero and neutral regions having positive D values. The main exception is the LCT gene, however, which we inferred to be selected from a *de novo* mutation, but which has a high D. The signal for SDN apparently comes more from the high EHH and low IHS values.

In general, our results are highly concordant with previous studies of these genes. Our estimates tend to gene, G6PD, we could not make any inferences, because we could not reproduce the observed pattern of diversity using simulations of positive directional selection. G6PD shows an extremely narrow region of reduced diversity, surrounded by a region of high diversity. This may be due to balancing selection between malaria resistance and reduced efficiency oxygen transport introducing a signal that cannot be reproduced by our simple model of directional selection. Alternatively, the X-linked mode of inheritance of this locus is not concordant with the assumptions of our model. This also highlights one of the dangers of ABC: It is crucial that the models investigated are able to reproduce the data observed; otherwise false inferences may be drawn. This danger inherent to any ABC approach is also highlighted by the fact that misidentification of the selected site will bias model choice results towards SSV (Figure S6). This can be explained by the fact that even if the neutral site is closely linked to the selected site, it is likely to “escape” the sweep by recombining away from the selected haplotype, thus giving the signal of selection from standing variation. Similarly, analyzing data simulated under a population bottleneck under a constant size model will bias the results towards stronger selection and SDN (Figure S7), presumably due to the younger age of mutations being taken as evidence of strong selection.

### Model choice accuracy

We have shown that it is much more difficult to estimate the model parameters *α*, and *t*_1_ from the SSV model than from the SDN model. This is unsurprising, as the SSV model has been shown to have a higher variance in allele age, which results in a higher expected variance for most summary statistics [8]. We further show that there is not enough information to estimate the initial frequency of the sweep *f*_1_. This is unsurprising, as the exact position of the switching point has likely only a minor effect on the data, especially as the effect of selection on the trajectory is weak when the allele frequency of the beneficial allele is low [27].

We further notice that the accuracy of our model choice procedure decreases when the signal of selection is weak. Consistent with previous findings, selection is very hard to detect if α is below about 100 [5], [11], [45]. This is also the point where our method gains power to distinguish between SVN from SDN. The initial frequency required to detect standing variation is moderate at around 3% for weak selection and 2% for stronger selection. However, selection has to be rather strong, at around *α* = 1,000 and initial frequencies have to be above 5% to allow accurate inference. Presumably, this is because below this threshold, the stochasticity of the trajectory is very large even under selection, and the difference between the two scenarios is small (see also Figure 1c). These findings are not particularly surprising, as selection scans based on summary statistics have been shown in general to have low power under these conditions [45].

These findings certainly limit the scope of our approach. Could we do better with a different strategy? As discussed in the introduction, the ABC approach simplifies data in two ways. First, instead of using the full data, we use an array of summary statistics. Second, we substitute an exact match between observation and simulations with an approximate match, depending on “close” simulations. Regarding the use of summary statistics, we note that summary statistics have been widely used to detect selection from genetic data [9]–[11], [14], [19], and currently provide the only way to detect selection from DNA sequence data. No full likelihood based method is available to detect selection from DNA sequence data that could be adapted to distinguish between the two sweep models entertained here.

The second simplification step is based on the number of simulations performed and the tolerance interval and is imposed by computational constraints. We examine the effect of different numbers of simulations and tolerance cutoffs on our results by calculating relative error rates of the posterior mean and the false negative rate of the model choice. We show in Table S1 that increasing the number of simulation by a large amount or changing the rejection parameter does not significantly improve our results, indicating that we do not lose a lot of information at this stage. This shows that the ABC approach reliably estimates the posterior based on the summary statistics, and as such use all the information available in these statistics. Statistics such as EHH, iHS, Tajima's *D*, etc, do not contain information that will allow us to provide more reliable estimates. It the light of this, it may appear disappointing that our method does not provide more accurate parameter estimates and more power to distinguish between models. However, it is important to realize that as previously argued, all information regarding selection is in the frequency path of the selected allele [26], [27]. For relatively small selection coefficients and/or small initial frequencies of the selected allele, the paths are very similar for the SSV and SDN models. Even if a full likelihood method could be developed, it is unlikely that it had much more power to distinguish between models.

A further simplification in our method is the restriction to a single population. Population differentiation measures, such as *F_ST_*, are one of the most successful ways to detect sweeps from standing variation [24], [86], and the inclusion of more realistic models of demography may improve our accuracy. Such models, however, require an additional estimation of multi-population demographic history, which greatly increases the complexity of the model.

While we applied our method only to human candidate loci, it should be possible to easily translate it to other species. In particular, as our simulation results suggest that we have more power to distinguish SDN and SSV if selection is strong, species with large population sizes, such as e.g. *Drosophila* or many microorganisms may be very promising targets for a similar study. Another possible target might be species with very strong artificial selection, such as domesticated animals or plants, where we may gain valuable insights on the domestication history of these species. Of course, our approach could also be combined with ancient DNA (e.g. [83]), which could provide much narrower confidence intervals on time estimates and also help improve estimates of selection coefficients.

The two selection models we consider here, the SSV and the SDN models, are nested models, Setting *f*_1_ = 1/2*N* in the SSV model recovers the SDN model. To facilitate Bayesian model choice we assign positive probability to *f*_1_ = 1/2*N*, and base our inferences on a choice between *f*_1_ = 1/2*N* and *f*_1_ ∼ U(0,0.2) (See Methods). ABC based model choice has recently been criticized and been shown to be biased in some cases where the statistics used are not sufficient [93], [94]. While some of the specific issues raised by [91] are not applicable in our setting because we consider nested models, we do not base our inference on sufficient statistics and the statistical properties of our model choice procedure are, therefore, largely unknown. To address this issue, and in general to validate our approach, we use a method introduced in [95]. We show in Figure S8 that our estimated probabilities only show bias for very small values of the Bayes factor, where there appears to be a bias towards inference of the SDN model for simulations generated under the SSV model with very low values of *f*_1_.

## Methods

### Models

In order to keep our problem simple, we condition on two important parameters: We assume that the exact site under selection is known from extraneous information, and we furthermore assume that the allele frequency *f*_cur_ of that site at the time of sampling, *t*_cur_ = 0 is known. The interpretation of the parameters is depicted graphically in Figure 1a.

Unless noted otherwise, we assume a panmictic diploid population of size *N* with an additive selection model where the ancestral homozygous, heterozygous and derived homozygous genotypes have fitness 1, 1+ *s/2* and 1 + *s*, respectively. However, the methodology applied here can easily be adapted to more complex scenarios, e.g. models involving multiple populations, more sophisticated demographic models, and other models of selection. For most simulated data sets, we will report the population scaled mutation rate *α = *4*Ns*, as the shape of the allele frequency trajectory depends only on that compound parameter [25]. However, for most of the genes we analyze previous estimates were made directly on *s* rather than the compound parameter. To facilitate comparisons, we report *s* for the genes we analyzed.

#### Sweep from a *de novo* mutation model

The sweep from a *de novo* mutation (SDN) models a single selective sweep and has two parameters: the mutation rate *μ*, and the selection coefficient *s*. For all simulations, we follow [80] and record the time *t*_0_ when the mutation arose, as *t*_0_ depends stochastically on *s*. The prior distributions we use for this model were *μ* ∼ U(0.5e-8,6e-8) and log_10_(*s*) ∼ U(−3,−0.5), where U is a uniform distribution.

#### Sweep from standing variation model

The sweep from standing variation (SSV) model is identical to the *de novo* mutation model, with the exception that we define a frequency *f*_1_ at which the mutation becomes selected. Unless noted otherwise, the priors for *μ* and *s* are the same as in the SDN model, and the prior for *f*_1_ is *f*_1_ ∼ U(0,0.2). In addition to *t*_0_, which is defined analogous to the SDN model, we are also interested in *t*_1_, the time when the mutation becomes selectively advantageous (i.e. the time when the mutation reaches frequency *f*_1_).

#### Neutral model

We also consider a neutral model (NT), without any selection. The only free parameter in this model is the mutation rate *μ*, with the same prior distribution as described under SDN model. As under the selection model, however, we still condition on one site having reached a final allele frequency of *f*_cur_, so this model does not correspond to the classical neutral coalescent.

### Approximate Bayesian Computation

We use a standard ABC approach [31], [32], using a post-sampling adjustment in the form of a GLM [96]. We used the *ABCToolbox* package [40], for specifying priors, rejection sampling and post-sampling adjustment. Unless specified otherwise, we perform 10^5^ simulations per model, and retained the 100 (0.1%) simulations with associated Euclidean distance between observed and simulated summary statistics closest to zero. To assess how the number of simulations and acceptance rates influence our results, we analyze 10,000 random data sets with up to 10^7^ simulations and varying acceptance rates. We show that these parameters have very little impact on the relative error for both the model choice and parameter estimates in Table S1.

#### Details of statistics used

We use a diverse array of summary statistics, with the goal of maximizing the information captured, while not incluing any statistics that just add noise. The statistics we used may be broadly classified into statistics based on haplotype patterns, and statistics based on the site frequency spectrum. The haplotype based statistics we used were iHS [19] and EHH [9]. We recorded EHH in a 10 kb, 20 kb and 50 kb window, centered on the selected site. For the SFS based statistics, we used Tajima's D [20], Fay & Wu's H [23], the average number of pairwise differences *π*, and the number of segregating sites *S* as statistics. All these statistics are calculated for three regions: A central region of 20 kb around the selected site, an intermediate region consisting of all sites 20–50 kb away from the selected site, and a faraway region consisting of all sites further than 50 kb away from the selected site. Following [97], we linearize all statistics using a Box-Cox-transformation [98]. To choose a set of informative summary statistics, we used a Partial Least Squares Discriminant Analysis (PLS-DA) [99], [100]. PLS-DA is a variant of Partial Least Squares regression, that, similarly to principal component analysis, extracts orthogonal components from a high-dimensional data set (in this case the summary statistics). In contrast to PCA, in PLS-DA these components are chosen such that the covariance between summary statistics and models is maximized [see e.g. 101]. We did our computations using the ‘plsda’ function of the mixOmics package for R, and kept the five first PLS-DA axes [100].

ABC has two crucial parameters independent of the model it is applied to: The number of simulations *n*_S_ and the acceptance rate *ε*. To assess the effect of these parameters on inference, we calculate the accuracy of our model choice estimates for various values of *n*_S_ and *ε* (Table S1).

### Simulations

All our data sets used for both the ABC inference and the assessment of our procedure are simulated using a modified version of the coalescent simulator *mbs* [102]. *Mbs* allows simulation of genetic data sets with a single selected site using the structured coalescent [26]. *mbs* first simulates the allele frequency trajectory of the site under selection, and then generates a data set conditional on that trajectory. We simulate allele frequency trajectories using Euler's method on the unscaled backwards diffusion equation with selection (eq 7.1 in [103]). This equation makes it very easy to incorporate population size changes by just changing the variance term. To simulate sweeps from standing variation, we set the selection coefficient (*s*) to zero the first time the trajectory reaches *f*_1_. To analyze simulated data sets, we generally simulate a 100 kb region with a recombination rate of 1.5 cM/Mb. For the human genes, we simulate the gene and a 50 kb flanking region on both sides, resulting in regions that are usually between 100 kb and 150 kb wide. Recombination rates and hotspots are modeled by using the HapMap recombination map [104] in the application to selected genes. For all simulated data sets we assume a constant-sized population. For the analysis of human genes, we use the population history estimated by [105]. Specific regions and details of the used regions are given in Table S2. To ensure that our method does not suffer from a high false positive rate, we also analyze regions 5 Mb downstream from the candidate genes, as they are presumably neutral. For three of genes (ASPM, G6PD, and PSCA), no data was available for these downstream regions, so we analyzed the remaining loci. Candidate loci for selection were chosen using the following criteria: i) they were required to have a derived allele frequency between 0.7 and 0.9 and ii) to be as closely to 5 Mb away from the actual candidate locus in the upstream gene as possible.

We estimate parameters from our models using the standard ABC procedure described above. The parameters we estimate are the mutation rate *μ*, the age of the sweep *t*_1_ the selection coefficient *s* and, only under the SSV model, the initial frequency *f*_1_ for the SSV model. In particular we want to determine if our posteriors are unbiased, and if we were able to get reasonable confidence in our estimates. To do this, we simulate data sets with fixed parameters and plotted the average posterior distribution for all parameters in Figure 2.

### Model choice

For model choice, our main goal is to calculate the relative probabilities of the models given the data, i.e. Pr(SSV | data), Pr(SDN | data) and Pr(NT | data), which we calculate using the marginal densities as proposed by [96]. To identify parameter regions where there is power to distinguish between the models, we simulate 1,000 data sets each under 30 different scenarios in three series, corresponding to three parameters of interest: The strength of selection *α*, the frequency when the mutation became selective advantageous *f*1 and *f*_cur_, the frequency at which the mutation is observed.

To test the algorithm for approximating Bayes factors, we also use a simulation approach. The estimator of the posterior probability from *k* simulations, 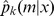, should have the property 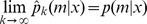 where *m* is a model indicator functions for a specific model. Also, for a particular draw from the posterior, *m* ^(0)^, we expect 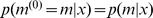, if the simulation algorithm works properly. In other words, 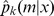, should asymptotically equal 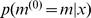, i.e. if 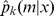 = *c*, we expect a proportion *c* of simulations to have been obtained from model *m*. Equivalently, for an estimated log Bayes factor, log_10_ = *c*, we expect a proportion 10*^c^*/(1+10*^c^*) of draws to be from model *m*. This prediction is tested in Figure S8, based on 10,000 random data sets from both the SDN and SSV model.

## Supporting Information

Figure S1 ROC plots. This figure gives ROC plots for the same data as in Figure 3. As we have three models, the first two columns compare both selection models with a neutral model, and the last two columns compare the two selection model, with the model better characterized as the null model plotted on the x-axis. The lines give the percentage of simulation assigned to the model on the y-axis (sensitivity), given a proportion of models assigned to the x-axis (specificity). Parameters used for the simulations are given above the plot and in the legend box.
(EPS)

Figure S2 Joint posteriors of *f*_1_ and *t*_1_ of simulations under the SDN when analyzed under the SSV model. Inferred joint posterior distribution of nine replicate simulation with parameters of *α* = 400, *μ* = 2.5e-8 are shown. Red and blank areas correspond to areas with zero probability, yellow areas indicate high probability densities. Notice that for most simulation the inferred initial frequency is below 2%.
(EPS)

Figure S3 Joint posteriors of *f*_1_ and *t*_1_ of simulations under the SSV when analyzed under the SSV model. Inferred joint posterior distribution of nine replicate simulation with parameters *f*_1_ = 0.1, of *α* = 400, *μ* = 2.5e-8 are shown. Red and blank areas correspond to areas with zero probability, yellow areas indicate high probability densities. Notice that for most simulation the inferred initial frequency is above 5%, but the inferred probability of *f*_1_ is often very inaccurate.
(EPS)

Figure S4 Joint posteriors of *f*_1_ and *t*_1_ for analyzed genes. Inferred joint posterior distribution of all seven genes analyzed in this paper. Red and blank areas correspond to areas with zero probability; yellow areas indicate high probability densities.
(EPS)

Figure S5 Observed summary statistic distributions. We show the observed untransformed summary statistics for all genes and genomic regions we analyzed in this study (see Table S2). Colors indicate the most likely mode of evolution: neutral evolution (grey), SDN (blue), SSV (orange) and undetermined (black). TD = Tajima's D, FWH = Fay and Wu's H. The suffix “global” indicates that the statistic was calculated for the entire gene, the suffix “close” indicates the statistic calculated on a 20kb window around the selected site.
(EPS)

Figure S6 Effect of misidentification of selected site: We show the posterior probabilities for SSV (orange), SDN (blue) and NT (grey) for simulations done from a *de novo* mutation(left panel) and standing variation (right panel), if we misidentify the selected allele. Simulations were done with selection strength α = 1,000, sample size *n* = 100, mutation rate *μ* = 2.5e-8 and recombination rate *ρ* = 3e-8. For the SSV simulation, *f*_1_ was set to 0.1 X-axes give the distance between the “true” selected allele from the site for which the summary statistics were calculated. If the distance is larger than 50 kb, we find a bias towards inferring SSV.
(EPS)

Figure S7 Bias in model choice due to a population bottleneck. We show the inferred posterior probabilities for SSV (orange), SDN (blue) and NT (grey) under a constant size model for simulations done under a bottleneck model. The bottleneck started 400 generations ago and lasted for 2,000 generations, which might be similar to the human out-of-Africa bottleneck Simulations were done with selection strength α = 1,000, sample size *n* = 100, mutation rate *μ* = 2.5e-8 and recombination rate *ρ* = 3e-8. For the SSV simulation, *f*_1_ was set to 0.1 X-axes give the strength of the bottleneck as a proportion of the current effective population size. Unaccounted demographic history results in a bias towards estimates of stronger selection.
(EPS)

Figure S8 Model choice bias. *B* denotes the Bayes factor in favor of the SSV model, *B* = Pr(SSV)/Pr(SDN). We simulated 10,000 data sets under both the SSV and SDN model, and performed our model choice procedure on each data set, and divided the distribution into discrete bins. The figure gives the observed (bars) and expected (red line) proportion of simulations from the SSV in each bin. As can be seen, there is a slight excess of simulations from the SSV on the lower end of the graph. The leftmost bin contains only 28 simulations, two of which were simulated under the SSV model. Both of these simulations had a *f*_1_ below 0.005, corresponding to a parameter region where the SSV and SDN models are very similar. The first and second row of numbers below the figure denote the number of simulations simulated under the SSV and SDN model, respectively.
(EPS)

Table S1 Relative Error for different numbers of simulations and acceptance rates. In this table, we give the relative error of the mean and the false negative rate of the model choice for 1000 data sets randomly simulated under the SSV model with varying number of simulation nSim and proportion of accepted simulations δ. FN = False negative rate in model choice. Chr: chromosome, pop: population we analyzed. All positions given are on the hg19 build of the human genome.
(DOCX)

Table S2 Details of genes and neutral regions analyzed in this study.
(DOCX)
