# Supplementary figures and images for: Distinguishing between Selective Sweeps from Standing Variation and from a De Novo Mutation
Source: PLoS Genet. 2012 Oct 11;8(10):e1003011. doi: 10.1371/journal.pgen.1003011 (PMC3469416; doi:10.1371/journal.pgen.1003011)

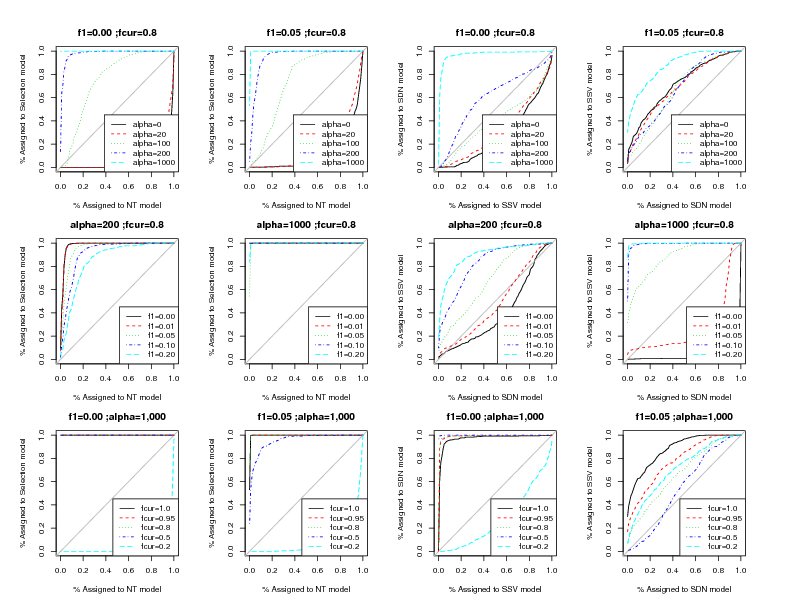

Supplement: Figure S1 — ROC plots. This figure gives ROC plots for the same data as in Figure 3. As we have three models, the first two columns compare both selection models with a neutral model, and the last two columns compare the two selection model, with the model better characterized as the null model plotted on the x-axis. The lines give the percentage of simulation assigned to the model on the y-axis (sensitivity), given a proportion of models assigned to the x-axis (specificity). Parameters used for the simulations are given above the plot and in the legend box. (EPS) [file pgen.1003011.s001.tif]

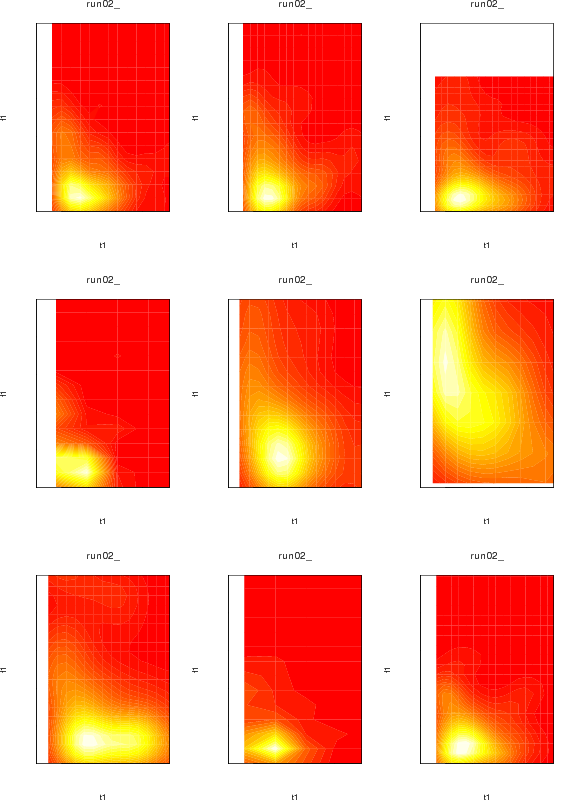

Supplement: Figure S2 — Joint posteriors of f 1 and t 1 of simulations under the SDN when analyzed under the SSV model. Inferred joint posterior distribution of nine replicate simulation with parameters of α = 400, μ = 2.5e-8 are shown. Red and blank areas correspond to areas with zero probability, yellow areas indicate high probability densities. Notice that for most simulation the inferred initial frequency is below 2%. (EPS) [file pgen.1003011.s002.tif]

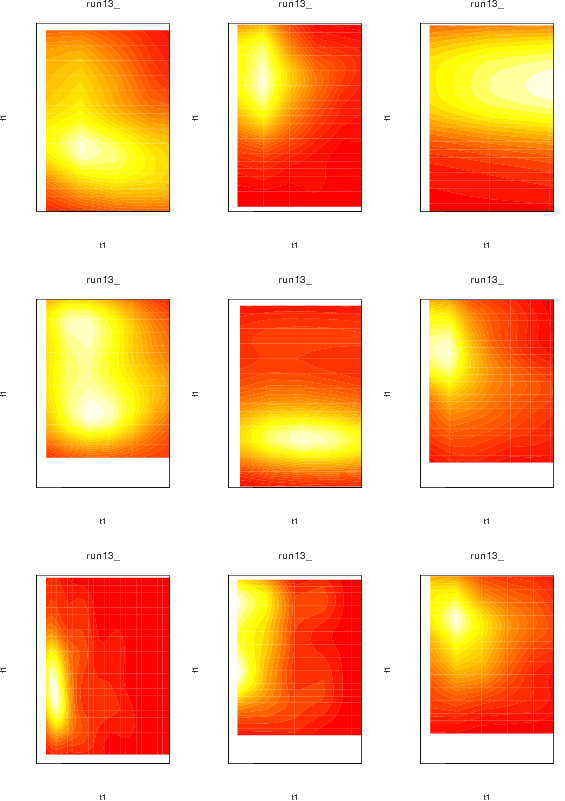

Supplement: Figure S3 — Joint posteriors of f 1 and t 1 of simulations under the SSV when analyzed under the SSV model. Inferred joint posterior distribution of nine replicate simulation with parameters f 1 = 0.1, of α = 400, μ = 2.5e-8 are shown. Red and blank areas correspond to areas with zero probability, yellow areas indicate high probability densities. Notice that for most simulation the inferred initial frequency is above 5%, but the inferred probability of f 1 is often very inaccurate. (EPS) [file pgen.1003011.s003.tif]

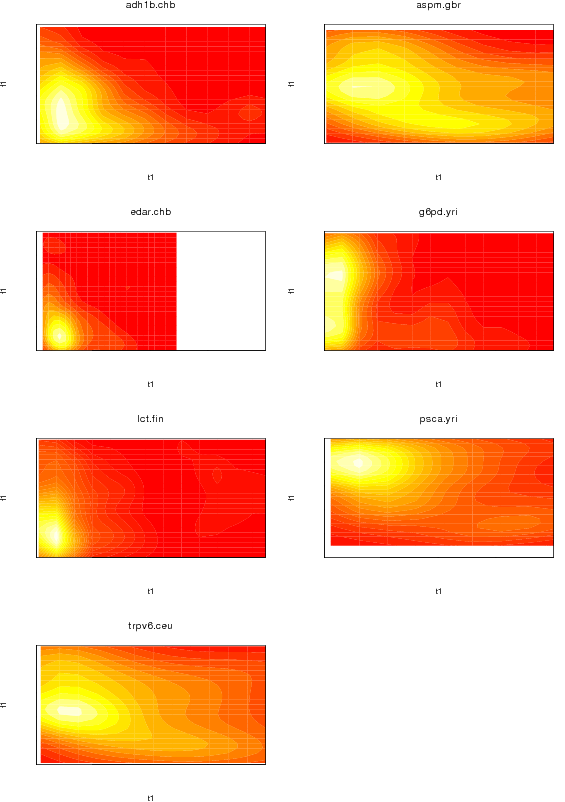

Supplement: Figure S4 — Joint posteriors of f 1 and t 1 for analyzed genes. Inferred joint posterior distribution of all seven genes analyzed in this paper. Red and blank areas correspond to areas with zero probability; yellow areas indicate high probability densities. (EPS) [file pgen.1003011.s004.tif]

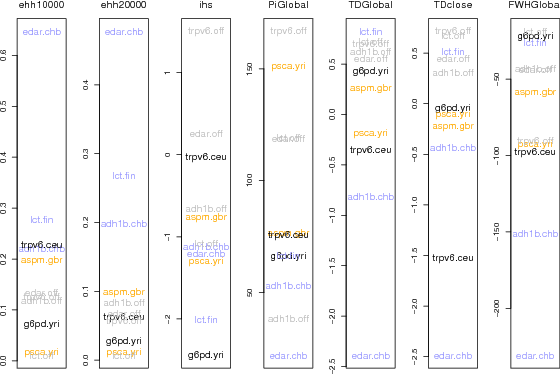

Supplement: Figure S5 — Observed summary statistic distributions. We show the observed untransformed summary statistics for all genes and genomic regions we analyzed in this study (see Table S2). Colors indicate the most likely mode of evolution: neutral evolution (grey), SDN (blue), SSV (orange) and undetermined (black). TD = Tajima's D, FWH = Fay and Wu's H. The suffix “global” indicates that the statistic was calculated for the entire gene, the suffix “close” indicates the statistic calculated on a 20kb window around the selected site. (EPS) [file pgen.1003011.s005.tif]

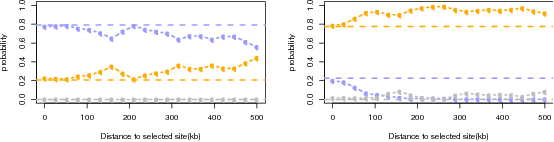

Supplement: Figure S6 — Effect of misidentification of selected site: We show the posterior probabilities for SSV (orange), SDN (blue) and NT (grey) for simulations done from a de novo mutation(left panel) and standing variation (right panel), if we misidentify the selected allele. Simulations were done with selection strength α = 1,000, sample size n = 100, mutation rate μ = 2.5e-8 and recombination rate ρ = 3e-8. For the SSV simulation, f 1 was set to 0.1 X-axes give the distance between the “true” selected allele from the site for which the summary statistics were calculated. If the distance is larger than 50 kb, we find a bias towards inferring SSV. (EPS) [file pgen.1003011.s006.tif]

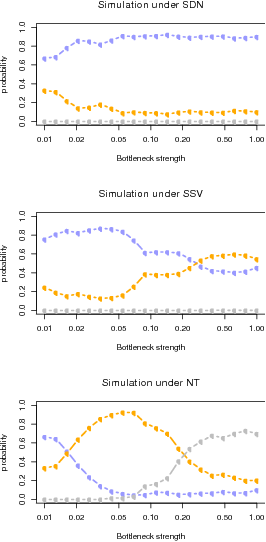

Supplement: Figure S7 — Bias in model choice due to a population bottleneck. We show the inferred posterior probabilities for SSV (orange), SDN (blue) and NT (grey) under a constant size model for simulations done under a bottleneck model. The bottleneck started 400 generations ago and lasted for 2,000 generations, which might be similar to the human out-of-Africa bottleneck Simulations were done with selection strength α = 1,000, sample size n = 100, mutation rate μ = 2.5e-8 and recombination rate ρ = 3e-8. For the SSV simulation, f 1 was set to 0.1 X-axes give the strength of the bottleneck as a proportion of the current effective population size. Unaccounted demographic history results in a bias towards estimates of stronger selection. (EPS) [file pgen.1003011.s007.tif]

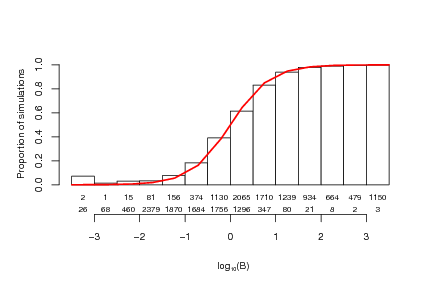

Supplement: Figure S8 — Model choice bias. B denotes the Bayes factor in favor of the SSV model, B = Pr(SSV)/Pr(SDN). We simulated 10,000 data sets under both the SSV and SDN model, and performed our model choice procedure on each data set, and divided the distribution into discrete bins. The figure gives the observed (bars) and expected (red line) proportion of simulations from the SSV in each bin. As can be seen, there is a slight excess of simulations from the SSV on the lower end of the graph. The leftmost bin contains only 28 simulations, two of which were simulated under the SSV model. Both of these simulations had a f 1 below 0.005, corresponding to a parameter region where the SSV and SDN models are very similar. The first and second row of numbers below the figure denote the number of simulations simulated under the SSV and SDN model, respectively. (EPS) [file pgen.1003011.s008.tif]
