# Supplementary material for: Distinguishing between Selective Sweeps from Standing Variation and from a De Novo Mutation
Source: PLoS Genet. 2012 Oct 11;8(10):e1003011. doi: 10.1371/journal.pgen.1003011 (PMC3469416; doi:10.1371/journal.pgen.1003011)
Supplement: Table S1 — Relative Error for different numbers of simulations and acceptance rates. In this table, we give the relative error of the mean and the false negative rate of the model choice for 1000 data sets randomly simulated under the SSV model with varying number of simulation nSim and proportion of accepted simulations δ. FN = False negative rate in model choice. Chr: chromosome, pop: population we analyzed. All positions given are on the hg19 build of the human genome. (DOCX) [file pgen.1003011.s009.docx]

| *n*_Sim_ | *δ* | RE(*f*_1_) | RE(log(*s*)) | RE(*µ*) | RE(log(*t*_1_)) | RE(log(*t*_0_)) | FN |
| --- | --- | --- | --- | --- | --- | --- | --- |
| 10^5^ | 10^-3^ | **0.0392** | **0.348** | **2.92e-9** | **0.366** | **0.321** | **0.258** |
| 10^5^ | 10^-2^ | **0.0387** | **0.348** | **2.91e-9** | **0.365** | **0.322** | **0.240** |
| 10^6^ | 10^-4^ | **0.0389** | **0.343** | **2.95e-9** | **0.361** | **0.321** | **0.250** |
| 10^6^ | 10^-3^ | **0.0388** | **0.345** | **2.93e-9** | **0.364** | **0.320** | **0.236** |
| 10^7^ | 10^-5^ | **0.0386** | **0.343** | **2.86e-9** | **0.361** | **0.323** | **0.258** |
| 10^7^ | 10^-4^ | **0.0387** | **0.344** | **2.97e-9** | **0.362** | **0.321** | **0.246** |
