# Supplementary material for: Distinguishing between Selective Sweeps from Standing Variation and from a De Novo Mutation
Source: PLoS Genet. 2012 Oct 11;8(10):e1003011. doi: 10.1371/journal.pgen.1003011 (PMC3469416; doi:10.1371/journal.pgen.1003011)
Supplement: Table S2 — Details of genes and neutral regions analyzed in this study. (DOCX) [file pgen.1003011.s010.docx]

|  |  |  |  |  |  | Selected site | |  |  |
| --- | --- | --- | --- | --- | --- | --- | --- | --- | --- |
| Gene | chr | function | freq | pop | Region sim | dbSNP id | Position | size | References |
| ADH1B | 4 | Alcohol metabolism | 0.71 | CHB | 100177528-100292572 | rs1229984 | 100239319 | 115044 | [50] |
| ASPM | 1 | microcephalism | 0.48 | GBR | 197007250-197165824 | rs41310927 | 197070697 | 158574 | [56] |
| EDAR | 2 | NF-κB Activation | 0.95 | CHB | 109460931-109605828 | rs3827760 | 109513601 | 144897 | [61] |
| G6PD | X | malaria resistance | 0.21 | YRI | 153709606-153825233 | rs1050828 | 153764217 | 115627 | [66] |
| LCT | 2 | lactase persistence | 0.56 | FIN | 136535946-136657220 | rs4988235 | 136608646 | 121274 | [73,74] |
| PSCA | 8 | Involved in bladder & pancreas cancer | 0.77 | YRI | 143691875-143834142 | rs2294008 | 143761931 | 142267 | [86] |
| TRPV6 | 7 | Calcium absorption | 0.91 | CEU | 142518960-142633477 | rs4987682 | 142569596 | 114517 | [90] |
| ADH1B.OFF | 4 | - | 0.71 | CHB | 105177528-105292572 | rs7672705 | 105247146 | 115044 |  |
| EDAR.OFF | 2 | - | 0.95 | CHB | 114460931-114605828 | rs34264290 | 114528334 | 144897 |  |
| LCT.OFF | 2 | - | 0.56 | FIN | 141535946-141657220 | rs59101965 | 141583482 | 121274 |  |
| TRPV6.OFF | 7 | - |  | CEU | 147518960-147633477 | . | 147583629 | 114517 |  |

Chr: chromosome, pop: population we analyzed. All positions given are on the hg19 build of the human genome.
